# Supplementary material for: Components of effective letters of recommendation: A cross-sectional survey of academic faculty
Source: PLoS One. 2024 Jan 23;19(1):e0296637. doi: 10.1371/journal.pone.0296637 (PMC10805295; doi:10.1371/journal.pone.0296637)
Supplement: S1 Appendix — (PDF) [file pone.0296637.s001.pdf]

Letters of Recommendation Survey

Q1: What is your gender?

- ☐ Female
- ☐ Male

Q2: Where did you complete your residency training?

- ☐ Asia
- ☐ Canada
- ☐ Europe (not UK)
- ☐ Middle East/North Africa
- ☐ anonymous
- ☐ United Kingdom
- ☐ United States
- ☐ Other: _________________

Q3: How many years have you been in practice (post residency/fellowship)?

- ☐ Range 0-30 years

Q4: What department are you working in?

- ☐ Anaesthesia/ Obstetrics and Gynaecology/ Surgery/ surgical subspecialty
- ☐ Family Medicine/ Internal Medicine/ medical subspecialty
- ☐ Paediatrics/ paediatric subspecialty
- ☐ Other: _________________

Q5: What is your current role?

- ☐ Associate program director or Program director
- ☐ Chair of Department or Chief of Division
- ☐ Clerkship Director
- ☐ Core teaching faculty
- ☐ Other: _________________

Q6: In an average year, how many letters of recommendations are you requested to write?

- None
- 1-5
- 6-10
- >10

Q7: What do you think is the purpose of a letter of recommendation?

- To help an applicant match into his/her desired program
- To provide an accurate assessment of the applicant
- A not-so-important part of the residency application process
- I don’t know

Q8: In your opinion, how important is a letter of recommendation?

- Not at all Important
- Somewhat Important
- Important
- Very Important

Q9: How long should a letter of recommendation be?

- One or two paragraphs is sufficient
- 1 Page is ideal
- 2 or more pages

Q10: Have you ever asked an applicant to write his/her own letter of recommendation?

- Yes
- No

Q11: Have you said no to a letter of recommendation request?

- Yes
- No

Q12: If yes, why?

- I am uncomfortable writing letters of recommendation
- I did not know applicant well

- ☐ I was too busy
- ☐ I would not have been able to write a positive/helpful letter for the applicant

Q13: Do you have a template for letters of recommendation?

- ☐ Yes
- ☐ No

Q14: Have you ever used the same letters of recommendation for different people?

- ☐ Yes
- ☐ No

Q15: Have you ever copied a letter of recommendation?

- ☐ Yes
- ☐ No

Q16: If yes, where did you copy them from?

- ☐ From a colleague?
- ☐ From online?

Q17: Do you include the following in your letters of recommendation?

A. Your academic background and years of experience
   - ☐ Never
   - ☐ Sometimes
   - ☐ Most of the times
   - ☐ Always

B. The nature of your relationship with the applicant
   - ☐ Never
   - ☐ Sometimes
   - ☐ Most of the times
   - ☐ Always

C. The duration of your relationship with the applicant
   - ☐ Never
   - ☐ Sometimes
   - ☐ Most of the times
   - ☐ Always

D. The applicant's qualifications/suitability for the position
   - ☐ Never
   - ☐ Sometimes

- ☐ Most of the times
- ☐ Always

E. The applicant’s research involvement
- ☐ Never
- ☐ Sometimes
- ☐ Most of the times
- ☐ Always

F. The applicant’s extracurricular activities
- ☐ Never
- ☐ Sometimes
- ☐ Most of the times
- ☐ Always

G. The applicant’s involvement in education
- ☐ Never
- ☐ Sometimes
- ☐ Most of the times
- ☐ Always

H. Specific examples of the applicant’s abilities and traits
- ☐ Never
- ☐ Sometimes
- ☐ Most of the times
- ☐ Always

I. Areas for improvement for the applicant
- ☐ Never
- ☐ Sometimes
- ☐ Most of the times
- ☐ Always

J. Global assessment of applicant (as compared to other applicants)
- ☐ Never
- ☐ Sometimes
- ☐ Most of the times
- ☐ Always

Q18: Do you comment on the following in letters of recommendations?

A. Work ethic
- ☐ Never
- ☐ Sometimes
- ☐ Most of the times
- ☐ Always

B. Intellectual curiosity
- ☐ Never
- ☐ Sometimes
- ☐ Most of the times
- ☐ Always

C. Medical knowledge
- ☐ Never
- ☐ Sometimes
- ☐ Most of the times
- ☐ Always

D. Problem solving and patient management
- ☐ Never
- ☐ Sometimes
- ☐ Most of the times
- ☐ Always

E. Behaviour and attitudinal skills
- ☐ Never
- ☐ Sometimes
- ☐ Most of the times
- ☐ Always

F. Communication skills
- ☐ Never
- ☐ Sometimes
- ☐ Most of the times
- ☐ Always

G. Ability to work in a team
- ☐ Never
- ☐ Sometimes
- ☐ Most of the times
- ☐ Always

H. Leadership
- ☐ Never
- ☐ Sometimes
- ☐ Most of the times
- ☐ Always

I. Motivation
- ☐ Never
- ☐ Sometimes
- ☐ Most of the times
- ☐ Always

J. Sense of responsibility
   - ☐ Never
   - ☐ Sometimes
   - ☐ Most of the times
   - ☐ Always

K. Procedural skills specific to the discipline
   - ☐ Never
   - ☐ Sometimes
   - ☐ Most of the times
   - ☐ Always

L. Patient advocacy
   - ☐ Never
   - ☐ Sometimes
   - ☐ Most of the times
   - ☐ Always

Q19: Are you aware of standardized letters of recommendation?

- ☐ Yes
- ☐ No

Q20: Are you aware of the residency programs that require standardized letters of recommendation?

- ☐ Yes
- ☐ No

Q21: Have you ever written a standardized letter of recommendation?

- ☐ Yes
- ☐ No

Q22: Have you ever received guidance/training in writing letters of recommendations?

- ☐ Yes
- ☐ No

Q23: If yes, where did you receive the training?

- ☐ Discussion with colleagues
- ☐ Discussion with program directors
- ☐ Faculty development sessions at your institution

- ☐ Journal articles
- ☐ Conferences
- ☐ Websites (e.g.AAMC, CORD)

Q24: Are you interested in receiving training on writing letters of recommendations?

- ☐ Yes
- ☐ No

Q25: If yes, which of the following would be your preferred method?

- ☐ Institution faculty development session
- ☐ Webinar
- ☐ Other: __________________
