# Supplementary material for: Components of effective letters of recommendation: A cross-sectional survey of academic faculty
Source: PLoS One. 2024 Jan 23;19(1):e0296637. doi: 10.1371/journal.pone.0296637 (PMC10805295; doi:10.1371/journal.pone.0296637)
Supplement: S2 Appendix — (PDF) [file pone.0296637.s002.pdf]

Status	What is your gender?	Where did you complete your residency training?	How many years have you been in practice? (post residency/post fellowship)	What department are you working in?	What is your current role?	In an average year, how many letters of recommendations are you requested to write?	What do you think is the purpose of a letter of recommendation?	In your opinion, how important is a letter of recommendation for an applicant's chances of matching into a residency or fellowship program?	How long should a letter of recommendation be?	Have you ever asked an applicant to write his/her own letter of recommendation?	Have you said no to a letter of recommendation request?	Why?	Do you have a template for letters of recommendation?
Complete	Male	United Kingdom	25	Pediatrics/Pediatric subspecialty	Core Teaching Faculty	1-5	To provide an accurate assessment of the applicant	Important	1 Page is ideal	No	No		No
Complete	Female	United States	20	Pediatrics/Pediatric subspecialty	Core Teaching Faculty	1-5	To provide an accurate assessment of the applicant	Very Important	1 Page is ideal	No	Yes	I was too busy	No
Incomplete													
Incomplete													
Incomplete	Male	United Kingdom	12	Anaesthesia/ Obstetrics and Gynecology/ Surgery/ Surgical Subspecialty	Core Teaching Faculty	1-5	To help an applicant match into his/her desired program	Very Important	1 Page is ideal	No	No		No
Complete	Female	UAE	10	Pediatrics/Pediatric subspecialty	Core Teaching Faculty	1-5	To help an applicant match into his/her desired program	Very Important	1 Page is ideal	No	No		No
Complete	Female	United States	21	Family Medicine/Internal Medicine/Medical Subspecialty	Associate Program Director or Program Director	1-5	To help an applicant match into his/her desired program	Very Important	1 Page is ideal	No	Yes	I did not know applicant well	No
Complete	Male	Canada	11	Pediatrics/Pediatric subspecialty	Core Teaching Faculty	1-5	To help an applicant match into his/her desired program	Very Important	1 Page is ideal	Yes	No		No
Complete	Female	United States	19	Family Medicine/Internal Medicine/Medical Subspecialty	Core Teaching Faculty	1-5	To help an applicant match into his/her desired program	Very Important	1 Page is ideal	No	Yes	I did not know applicant well	No
Complete	Male	United Kingdom	13	Anaesthesia/ Obstetrics and Gynecology/ Surgery/ Surgical Subspecialty	Core Teaching Faculty	1-5	To help an applicant match into his/her desired program	Very Important	1 Page is ideal	No	Yes	I was too busy	No
Complete	Female	United States	20	Family Medicine/Internal Medicine/Medical Subspecialty	Associate Program Director or Program Director	1-5	To provide an accurate assessment of the applicant	Very Important	1 Page is ideal	No	Yes	I did not know applicant well	No
Incomplete	Female	United States	20	Family Medicine/Internal Medicine/Medical Subspecialty	Associate Program Director or Program Director								
Complete	Female	UAE	8	Anaesthesia/ Obstetrics and Gynecology/ Surgery/ Surgical Subspecialty	Core Teaching Faculty	1-5	To help an applicant match into his/her desired program	Very Important	1 Page is ideal	No	Yes	I did not know applicant well	No
Complete	Male	United Kingdom	19	Anaesthesia/ Obstetrics and Gynecology/ Surgery/ Surgical Subspecialty	Core Teaching Faculty	1-5	To help an applicant match into his/her desired program	Very Important	1 Page is ideal	No	No		No
Complete	Female	UAE	7	Family Medicine/Internal Medicine/Medical Subspecialty	Core Teaching Faculty	1-5	To help an applicant match into his/her desired program	Very Important	1 Page is ideal	No	Yes	I did not know applicant well	No
Complete	Male	Canada	12	Anaesthesia/ Obstetrics and Gynecology/ Surgery/ Surgical Subspecialty	Core Teaching Faculty	1-5	To help an applicant match into his/her desired program	Very Important	1-2 pages	No	Yes	I did not know applicant well	No
Complete	Female	United States	20	Family Medicine/Internal Medicine/Medical Subspecialty	Core Teaching Faculty	1-5	To help an applicant match into his/her desired program	Very Important	1 Page is ideal	No	Yes	I was too busy	No
Complete	Female	UAE	7	Family Medicine/Internal Medicine/Medical Subspecialty	Core Teaching Faculty	1-5	To help an applicant match into his/her desired program	Very Important	1 Page is ideal	No	Yes	I was too busy	No
Complete	Male	UAE	7	Family Medicine/Internal Medicine/Medical Subspecialty	Core Teaching Faculty	1-5	To help an applicant match into his/her desired program	Very Important	1 Page is ideal	No	Yes	I was too busy	No
Complete	Female	UAE	7	Anaesthesia/ Obstetrics and Gynecology/ Surgery/ Surgical Subspecialty	Core Teaching Faculty	1-5	To help an applicant match into his/her desired program	Very Important	1 Page is ideal	No	No		No
Complete	Female	Asia	9	Pediatrics/Pediatric subspecialty	Core Teaching Faculty	1-5	To help an applicant match into his/her desired program	Very Important	1 Page is ideal	No	No		No
Complete	Male	Canada	12	Family Medicine/Internal Medicine/Medical Subspecialty	Core Teaching Faculty	1-5	To help an applicant match into his/her desired program	Very Important	1 Page is ideal	No	Yes	I was too busy	No
Complete	Female	Canada	13	Anaesthesia/ Obstetrics and Gynecology/ Surgery/ Surgical Subspecialty	Core Teaching Faculty	1-5	To help an applicant match into his/her desired program	Very Important	1 Page is ideal	No	Yes	I was too busy	No
Complete	Female	United States	18	Family Medicine/Internal Medicine/Medical Subspecialty	Core Teaching Faculty	1-5	To help an applicant match into his/her desired program	Very Important	1 Page is ideal	No	Yes	I did not know applicant well	No
Complete	Male	United Kingdom	13	Family Medicine/Internal Medicine/Medical Subspecialty	Core Teaching Faculty	1-5	To help an applicant match into his/her desired program	Very Important	1 Page is ideal	No	Yes	I was too busy	No
Complete	Male	United Kingdom	14	Family Medicine/Internal Medicine/Medical Subspecialty	Chief of Division or Chair of Department	1-5	To help an applicant match into his/her desired program	Very Important	1-2 pages	No	Yes	I was too busy	No
Complete	Male	United Kingdom	7	Anaesthesia/ Obstetrics and Gynecology/ Surgery/ Surgical Subspecialty	Core Teaching Faculty	1-5	To help an applicant match into his/her desired program	Very Important	1 Page is ideal	No	Yes	I was too busy	No
Complete	Male	Canada	5	Pediatrics/Pediatric subspecialty	Core Teaching Faculty	6-10	To help an applicant match into his/her desired program	Very Important	1 Page is ideal	No	Yes	I was too busy	No
Incomplete	Male	Canada	7	Pediatrics/Pediatric subspecialty	Core Teaching Faculty								
Complete	Male	United States	20	Anaesthesia/ Obstetrics and Gynecology/ Surgery/ Surgical Subspecialty	Chief of Division or Chair of Department	1-5	To help an applicant match into his/her desired program	Very Important	1-2 pages	No	Yes	I was too busy	No
Complete	Female	United States	22	Family Medicine/Internal Medicine/Medical Subspecialty	Chief of Division or Chair of Department	1-5	To help an applicant match into his/her desired program	Very Important	1-2 pages	No	Yes	I was too busy	No
Complete	Female	United States	10	Pediatrics/Pediatric subspecialty	Core Teaching Faculty	1-5	To help an applicant match into his/her desired program	Very Important	1 Page is ideal	No	Yes	I did not know applicant well	Yes
Complete	Female	UAE	4	Family Medicine/Internal Medicine/Medical Subspecialty	Core Teaching Faculty	1-5	A not-so-important part of the residency application process	Somewhat Important	1 Page is ideal	No	Yes	I did not know applicant well	No
Complete	Female	UAE	7	Pediatrics/Pediatric subspecialty	Associate Program Director or Program Director	1-5	To help an applicant match into his/her desired program	Somewhat Important	1 Page is ideal	No	Yes	I would not have been able to write a positive/helpful letter for the applicant	No
Complete	Female	United States	20	Family Medicine/Internal Medicine/Medical Subspecialty	Associate Program Director or Program Director	6-10	To help an applicant match into his/her desired program	Very Important	1 Page is ideal	No	No		No
Complete	Female	United States	20	Family Medicine/Internal Medicine/Medical Subspecialty	Associate Program Director or Program Director	1-5	To help an applicant match into his/her desired program	Very Important	1 Page is ideal	No	Yes	I did not know applicant well	No
Complete	Male	Canada	13	Family Medicine/Internal Medicine/Medical Subspecialty	Core Teaching Faculty	1-5	To help an applicant match into his/her desired program	Very Important	1 Page is ideal	No	Yes	I did not know applicant well	No
Complete	Male	United Kingdom	12	Pediatrics/Pediatric subspecialty	Core Teaching Faculty	1-5	To help an applicant match into his/her desired program	Important	1 Page is ideal	No	No		No
Complete	Male	Canada	14	Pediatrics/Pediatric subspecialty	Chief of Division or Chair of Department	6-10	To help an applicant match into his/her desired program	Important	1 Page is ideal	No	No		No

Status	What is your gender?	Where did you complete your residency training?	How many years have you been in practice? (post residency/post fellowship)	What department are you working in?	What is your current role?	In an average year, how many letters of recommendations are you requested to write?	What do you think is the purpose of a letter of recommendation?	In your opinion, how important is a letter of recommendation for an applicant's chances of matching into a residency or fellowship program?	How long should a letter of recommendation be?	Have you ever asked an applicant to write his/her own letter of recommendation?	Have you said no to a letter of recommendation request?	Why?	Do you have a template for letters of recommendation?
Complete	Female	United Kingdom	10	Anaesthesia/ Obstetrics and Gynecology/ Surgery/ Surgical Subspecialty	Core Teaching Faculty	1-5	To provide an accurate assessment of the applicant	Important	1-2 pages	No	Yes	I would not have been able to write a positive/helpful letter for the applicant	Yes
Incomplete	Male	Canada	13	Anaesthesia/ Obstetrics and Gynecology/ Surgery/ Surgical Subspecialty	Core Teaching Faculty	1-5	To help an applicant match into his/her desired program	Important	1 Page is ideal	No	No		No
Incomplete	Male	Canada	14	Anaesthesia/ Obstetrics and Gynecology/ Surgery/ Surgical Subspecialty	Core Teaching Faculty								
Complete	Male	Canada	10	Anaesthesia/ Obstetrics and Gynecology/ Surgery/ Surgical Subspecialty	Core Teaching Faculty	1-5	To help an applicant match into his/her desired program	Important	1 Page is ideal	No	No		No
Complete	Male	United Kingdom	10	Anaesthesia/ Obstetrics and Gynecology/ Surgery/ Surgical Subspecialty	Core Teaching Faculty	1-5	To help an applicant match into his/her desired program	Important	1 Page is ideal	No	No		No
Complete	Male	Europe (not UK)	11	Anaesthesia/ Obstetrics and Gynecology/ Surgery/ Surgical Subspecialty	Core Teaching Faculty	1-5	To help an applicant match into his/her desired program	Important	1 Page is ideal	No	Yes	I did not know applicant well	No
Incomplete													
Complete	Male	Canada	9	Anaesthesia/ Obstetrics and Gynecology/ Surgery/ Surgical Subspecialty	Core Teaching Faculty	1-5	To help an applicant match into his/her desired program	Important	1 Page is ideal	No	Yes	I am uncomfortable writing letters of recommendation	No
Complete	Female	Europe (not UK)	10	Other, please describe: Ophthalmology	Other, please describe Medical doctor	1-5	To help an applicant match into his/her desired program	Very Important	1 Page is ideal	No	No		No
Complete	Female	Europe (not UK)	20	Pediatrics/Pediatric subspecialty	Chief of Division or Chair of Department	6-10	To provide an accurate assessment of the applicant	Very Important	1 Page is ideal	Yes	No		Yes
Complete	Male	United Kingdom	15	Anaesthesia/ Obstetrics and Gynecology/ Surgery/ Surgical Subspecialty	Chief of Division or Chair of Department	1-5	To help an applicant match into his/her desired program	Important	1 Page is ideal	No	Yes	I did not know applicant well	No
Complete	Male	Europe (not UK)	8	Anaesthesia/ Obstetrics and Gynecology/ Surgery/ Surgical Subspecialty	Associate Program Director or Program Director	1-5	To help an applicant match into his/her desired program	Somewhat Important	1 Page is ideal	No	No		No
Complete	Male	UAE	11	Family Medicine/Internal Medicine/Medical Subspecialty	Core Teaching Faculty	1-5	To help an applicant match into his/her desired program	Somewhat Important	1 Page is ideal	No	No		No
Incomplete	Female	Asia	24	Family Medicine/Internal Medicine/Medical Subspecialty	Other, please describe								
Incomplete													
Complete	Male	Asia	28	Family Medicine/Internal Medicine/Medical Subspecialty	Core Teaching Faculty	1-5	To help an applicant match into his/her desired program	Somewhat Important	1 Page is ideal	Yes	No		No
Complete	Male	United States	8	Family Medicine/Internal Medicine/Medical Subspecialty	Core Teaching Faculty	6-10	To provide an accurate assessment of the applicant	Important	1 Page is ideal	Yes	No		No
Incomplete													
Incomplete													
Complete	Female	UAE	18	Family Medicine/Internal Medicine/Medical Subspecialty	Chief of Division or Chair of Department	>10	To help an applicant match into his/her desired program	Important	1 Page is ideal	No	No		Yes
Incomplete													
Incomplete													
Incomplete													
Complete	Female	United States	17	Family Medicine/Internal Medicine/Medical Subspecialty	Core Teaching Faculty	1-5	To help an applicant match into his/her desired program	Important	1 Page is ideal	No	No		Yes
Complete	Male	United States	29	Other, please describe: Ophthalmolgy	Chief of Division or Chair of Department	6-10	To help an applicant match into his/her desired program	Somewhat Important	1 Page is ideal	No	Yes	I would not have been able to write a positive/helpful letter for the applicant	No
Complete	Male	Asia	5	Other, please describe: Cardiology	Other, please describe Associate Staff Physician	1-5	To help an applicant match into his/her desired program	Very Important	1-2 pages	No	No		Yes
Complete	Female	United States	6	Other, please describe: Emergency medicine	Core Teaching Faculty	1-5	To provide an accurate assessment of the applicant	Important	1 Page is ideal	No	No		Yes
Complete	Male	Middle East/North Africa (Not UAE)	10	Anaesthesia/ Obstetrics and Gynecology/ Surgery/ Surgical Subspecialty	Core Teaching Faculty	1-5	To provide an accurate assessment of the applicant	Very Important	1 Page is ideal	No	No		Yes
Incomplete													
Incomplete													
Complete	Male	United States	14	Family Medicine/Internal Medicine/Medical Subspecialty	Associate Program Director or Program Director	6-10	To provide an accurate assessment of the applicant	Important	1 Page is ideal	No	Yes	I would not have been able to write a positive/helpful letter for the applicant	Yes
Complete	Male	United States	13	Family Medicine/Internal Medicine/Medical Subspecialty	Core Teaching Faculty	1-5	To help an applicant match into his/her desired program	Very Important	1 Page is ideal	No	No		No
Complete	Male	Middle East/North Africa (Not UAE)	9	Family Medicine/Internal Medicine/Medical Subspecialty	Core Teaching Faculty	None	To help an applicant match into his/her desired program	Somewhat Important	1 Page is ideal	No	No		No
Complete	Male	Asia	8	Family Medicine/Internal Medicine/Medical Subspecialty	Core Teaching Faculty	1-5	To help an applicant match into his/her desired program	Important	1 Page is ideal	No	Yes	I did not know applicant well	No
Complete	Male	Asia	5	Family Medicine/Internal Medicine/Medical Subspecialty	Core Teaching Faculty	1-5	To provide an accurate assessment of the applicant	Very Important	1-2 pages	No	No		No
Incomplete													
Incomplete	Male	United States	1	Family Medicine/Internal Medicine/Medical Subspecialty	Other, please describe Faculty								
Incomplete													
Incomplete													
Complete	Male	United States	14	Other, please describe: Emergency medicine	Associate Program Director or Program Director	1-5	A not-so-important part of the residency application process	Not at all Important	1 Page is ideal	No	Yes	I did not know applicant well	Yes
Complete	Male	Canada	7	Anaesthesia/ Obstetrics and Gynecology/ Surgery/ Surgical Subspecialty	Associate Program Director or Program Director	1-5	To help an applicant match into his/her desired program	Somewhat Important	1 Page is ideal	No	Yes	I would not have been able to write a positive/helpful letter for the applicant	Yes
Incomplete													
Incomplete													

Status	What is your gender?	Where did you complete your residency training?	How many years have you been in practice? (post residency/post fellowship)	What department are you working in?	What is your current role?	In an average year, how many letters of recommendations are you requested to write?	What do you think is the purpose of a letter of recommendation?	In your opinion, how important is a letter of recommendation for an applicant's chances of matching into a residency or fellowship program?	How long should a letter of recommendation be?	Have you ever asked an applicant to write his/her own letter of recommendation?	Have you said no to a letter of recommendation request?	Why?	Do you have a template for letters of recommendation?
Complete	Female	Europe (not UK)	17	Anaesthesia/ Obstetrics and Gynecology/ Surgery/ Surgical Subspecialty	Core Teaching Faculty	1-5	Other, please describe To highlight the candidate strengths and achievements that are not so visible in a CV	Important	1 Page is ideal	Yes	Yes	I would not have been able to write a positive/helpful letter for the applicant	No
Complete	Male	Europe (not UK)	25	Other, please describe: Eye	Core Teaching Faculty	6-10	To provide an accurate assessment of the applicant	Very Important	1 Page is ideal	No	Yes	I would not have been able to write a positive/helpful letter for the applicant	No
Complete	Male	Canada	18	Anaesthesia/ Obstetrics and Gynecology/ Surgery/ Surgical Subspecialty	Core Teaching Faculty	1-5	To help an applicant match into his/her desired program	Important	1 Page is ideal	No	Yes	I was too busy	No
Complete	Male	Europe (not UK)	4	Anaesthesia/ Obstetrics and Gynecology/ Surgery/ Surgical Subspecialty	Other, please describe Specialist	None	To help an applicant match into his/her desired program	Somewhat Important	One or two paragraphs is sufficient	No	No		No
Complete	Female	United States	9	Anaesthesia/ Obstetrics and Gynecology/ Surgery/ Surgical Subspecialty	Associate Program Director or Program Director	1-5	To help an applicant match into his/her desired program	Very Important	One or two paragraphs is sufficient	No	Yes	I would not have been able to write a positive/helpful letter for the applicant	No
Complete	Male	United States	12	Anaesthesia/ Obstetrics and Gynecology/ Surgery/ Surgical Subspecialty	Core Teaching Faculty	1-5	To help an applicant match into his/her desired program	Important	1 Page is ideal	No	Yes	I was too busy	No
Complete	Female	Middle East/North Africa (Not UAE)	10	Anaesthesia/ Obstetrics and Gynecology/ Surgery/ Surgical Subspecialty	Other, please describe	None	To provide an accurate assessment of the applicant	Important	One or two paragraphs is sufficient	No	No		No
Complete	Male	Asia	20	Anaesthesia/ Obstetrics and Gynecology/ Surgery/ Surgical Subspecialty	Core Teaching Faculty	None	I don't know	Somewhat Important	One or two paragraphs is sufficient	No	No		No
Complete	Male	United States	11	Anaesthesia/ Obstetrics and Gynecology/ Surgery/ Surgical Subspecialty	Associate Program Director or Program Director	>10	To provide an accurate assessment of the applicant	Important	1 Page is ideal	No	Yes	I would not have been able to write a positive/helpful letter for the applicant	Yes
Complete	Male	United Kingdom	8	Other, please describe: Emergency Medicine	Core Teaching Faculty	1-5	To help an applicant match into his/her desired program	Important	1 Page is ideal	No	Yes	I would not have been able to write a positive/helpful letter for the applicant	No
Incomplete	Male	United States	22	Other, please describe: Ophthalmology	Other, please describe consultant								
Complete	Male	Asia	21	Anaesthesia/ Obstetrics and Gynecology/ Surgery/ Surgical Subspecialty	Associate Program Director or Program Director	1-5	To help an applicant match into his/her desired program	Important	1 Page is ideal	Yes	Yes	I did not know applicant well	No
Complete	Male	United States	22	Anaesthesia/ Obstetrics and Gynecology/ Surgery/ Surgical Subspecialty	Clerkship Director	>10	To provide an accurate assessment of the applicant	Somewhat Important	1 Page is ideal	No	No		No
Complete	Male	United States	10	Other, please describe: Critical Care	Associate Program Director or Program Director	1-5	To help an applicant match into his/her desired program	Important	1 Page is ideal	Yes	Yes	I would not have been able to write a positive/helpful letter for the applicant	No
Complete	Male	United States	21	Family Medicine/Internal Medicine/Medical Subspecialty	Associate Program Director or Program Director	1-5	To help an applicant match into his/her desired program	Somewhat Important	1-2 pages	No	Yes	I did not know applicant well	No
Incomplete	Male	United States	12	Family Medicine/Internal Medicine/Medical Subspecialty	Core Teaching Faculty	1-5	To provide an accurate assessment of the applicant	Somewhat Important	1 Page is ideal	No	Yes	I would not have been able to write a positive/helpful letter for the applicant	Yes
Complete	Male	Canada	16	Other, please describe: ED	Chief of Division or Chair of Department	6-10	To provide an accurate assessment of the applicant	Important	1 Page is ideal	No	Yes	I did not know applicant well	Yes
Complete	Male	United States	17	Anaesthesia/ Obstetrics and Gynecology/ Surgery/ Surgical Subspecialty	Core Teaching Faculty	1-5	To help an applicant match into his/her desired program	Very Important	1 Page is ideal	No	Yes	I was too busy	No
Complete	Male	United States	27	Other, please describe: Ophthalmology	Chief of Division or Chair of Department	1-5	Other, please describe Job, fellowship or residency position. Faculty Promotion.	Somewhat Important	1 Page is ideal	Yes	No		No
Complete	Male	United States	24	Other, please describe: imaging	Associate Program Director or Program Director	1-5	To provide an accurate assessment of the applicant	Important	1 Page is ideal	No	No		No
Complete	Male	United States	14	Anaesthesia/ Obstetrics and Gynecology/ Surgery/ Surgical Subspecialty	Associate Program Director or Program Director	1-5	To provide an accurate assessment of the applicant	Important	1 Page is ideal	No	No		Yes
Incomplete	Male	United States	20	Other, please describe: oncology	Associate Program Director or Program Director								
Complete	Male	Middle East/North Africa (Not UAE)	9	Anaesthesia/ Obstetrics and Gynecology/ Surgery/ Surgical Subspecialty	Associate Program Director or Program Director	1-5	To help an applicant match into his/her desired program	Important	1 Page is ideal	No	No		No
Complete	Male	United States	27	Other, please describe: Ophthalmology	Core Teaching Faculty	1-5	To provide an accurate assessment of the applicant	Important	1 Page is ideal	No	Yes	I did not know applicant well	No
Complete	Male	United States	24	Family Medicine/Internal Medicine/Medical Subspecialty	Other, please describe Consultant Hospitalist	1-5	To provide an accurate assessment of the applicant	Very Important	1 Page is ideal	No	Yes	I would not have been able to write a positive/helpful letter for the applicant	No
Complete	Male	United States	13	Anaesthesia/ Obstetrics and Gynecology/ Surgery/ Surgical Subspecialty	Associate Program Director or Program Director	1-5	To provide an accurate assessment of the applicant	Important	1 Page is ideal	No	No		Yes
Complete	Male	Europe (not UK)	10	Other, please describe: Ophthalmology	Associate Program Director or Program Director	1-5	To provide an accurate assessment of the applicant	Important	1 Page is ideal	No	No		Yes
Complete	Male	Asia	16	Anaesthesia/ Obstetrics and Gynecology/ Surgery/ Surgical Subspecialty	Other, please describe Clinical Preceptor and Academic Activity Director	1-5	To provide an accurate assessment of the applicant	Very Important	1-2 pages	No	Yes	I did not know applicant well	No

Status	What is your gender?	Where did you complete your residency training?	How many years have you been in practice? (post residency/post fellowship)	What department are you working in?	What is your current role?	In an average year, how many letters of recommendations are you requested to write?	What do you think is the purpose of a letter of recommendation?	In your opinion, how important is a letter of recommendation for an applicant's chances of matching into a residency or fellowship program?	How long should a letter of recommendation be?	Have you ever asked an applicant to write his/her own letter of recommendation?	Have you said no to a letter of recommendation request?	Why?	Do you have a template for letters of recommendation?
Complete	Male	United Kingdom	30	Family Medicine/Internal Medicine/Medical Subspecialty	Core Teaching Faculty	6-10	To provide an accurate assessment of the applicant	Very Important	1 Page is ideal	No	No		Yes
Complete	Female	United Kingdom	12	Family Medicine/Internal Medicine/Medical Subspecialty	Associate Program Director or Program Director	1-5	To help an applicant match into his/her desired program	Somewhat Important	1 Page is ideal	No	No		No
Complete	Male	United Kingdom	29	Family Medicine/Internal Medicine/Medical Subspecialty	Other, please describe	>10	To help an applicant match into his/her desired program	Somewhat Important	One or two paragraphs is sufficient	No	No		Yes
Complete	Male	United Kingdom	18	Family Medicine/Internal Medicine/Medical Subspecialty	Chief of Division or Chair of Department	1-5	To provide an accurate assessment of the applicant	Important	1-2 pages	No	No		No
Complete	Male	United Kingdom	14	Family Medicine/Internal Medicine/Medical Subspecialty	Associate Program Director or Program Director	>10	To help an applicant match into his/her desired program	Very Important	1 Page is ideal	No	No		No
Complete	Male	United Kingdom	20	Family Medicine/Internal Medicine/Medical Subspecialty	Core Teaching Faculty	1-5	To help an applicant match into his/her desired program	Somewhat Important	1 Page is ideal	No	No		Yes
Complete	Female	UAE	5	Other, please describe: Radiology	Associate Program Director or Program Director	1-5	To help an applicant match into his/her desired program	Important	1-2 pages	No	No		No
Incomplete													
Complete	Male	Middle East/North Africa (Not UAE)	20	Pediatrics/Pediatric subspecialty	Chief of Division or Chair of Department	>10	Other, please describe Many purposes, including match a program, job application, fellow ship application and assessment	Very Important	1 Page is ideal	No	Yes	Other Both not knowing the applicant and no positive letter	Yes
Incomplete	Male	United States	13	Other, please describe: .	Core Teaching Faculty								
Incomplete	Female	United States	20	Family Medicine/Internal Medicine/Medical Subspecialty	Associate Program Director or Program Director								
Complete	Female	United States	23	Family Medicine/Internal Medicine/Medical Subspecialty	Core Teaching Faculty	1-5	To provide an accurate assessment of the applicant	Very Important	1 Page is ideal	No	Yes	I did not know applicant well	No
Complete	Male	United Kingdom	25	Pediatrics/Pediatric subspecialty	Core Teaching Faculty	1-5	To provide an accurate assessment of the applicant	Very Important	1 Page is ideal	No	No		No
Complete	Male	United Kingdom	14	Family Medicine/Internal Medicine/Medical Subspecialty	Core Teaching Faculty	1-5	To help an applicant match into his/her desired program	Somewhat Important	One or two paragraphs is sufficient	No	No		No
Complete	Male	United Kingdom	12	Other, please describe: radiology	Core Teaching Faculty	None	To provide an accurate assessment of the applicant	Somewhat Important	One or two paragraphs is sufficient	No	No		No
Complete	Male	United States	22	Pediatrics/Pediatric subspecialty	Core Teaching Faculty	6-10	To help an applicant match into his/her desired program	Somewhat Important	1-2 pages	No	Yes	I would not have been able to write a positive/helpful letter for the applicant	No
Complete	Male	United Kingdom	11	Other, please describe: ;-)	Core Teaching Faculty	6-10	To help an applicant match into his/her desired program	Somewhat Important	One or two paragraphs is sufficient	No	No		No
Incomplete	Female	United States	22	Other, please describe: Radiology	Chief of Division or Chair of Department								
Incomplete	Female	UAE	21	Family Medicine/Internal Medicine/Medical Subspecialty	Other, please describe	1-5	To help an applicant match into his/her desired program	Somewhat Important	1 Page is ideal	Yes	No		Yes

Have you ever used the same letters of recommendation for different people?	Have you ever copied a letter of recommendation?	Where did you copy the letter(s) of recommendation from?	Your academic background and years of experience (Do you include the following in your letters of recommendation?)	The nature of your relationship with the applicant (Do you include the following in your letters of recommendation?)	The duration of your relationship with the applicant (Do you include the following in your letters of recommendation?)	The applicant's qualifications/suitability for the position (Do you include the following in your letters of recommendation?)	The applicant's research involvement (Do you include the following in your letters of recommendation?)	The applicant's extracurricular activities (Do you include the following in your letters of recommendation?)	The applicant's involvement in education (Do you include the following in your letters of recommendation?)	Specific examples of the applicant's abilities and traits (Do you include the following in your letters of recommendation?)	Areas for improvement for the applicant (Do you include the following in your letters of recommendation?)	Global assessment of applicant (as compared to other applicants) (Do you include the following in your letters of recommendation?)	Work ethic (Do you comment on the following in letters of recommendations?)
No	No		Sometimes	Always	Always	Most of the times	Most of the times	Most of the times	Most of the times	Sometimes	Sometimes	Most of the times	Most of the times
Yes	No		Most of the times	Most of the times	Most of the times	Sometimes	Never	Never	Never	Never	Never	Most of the times	Never
No	No		Always	Always	Most of the times	Most of the times	Sometimes	Sometimes	Sometimes	Sometimes	Never	Most of the times	Sometimes
No	No		Sometimes	Sometimes	Sometimes	Sometimes	Never	Never	Never	Sometimes	Never	Most of the times	Never
Yes	No		Always	Always	Always	Sometimes	Never	Never	Never	Never	Never	Always	Never
Yes	No		Always	Always	Sometimes	Sometimes	Never	Never	Never	Never	Never	Sometimes	Sometimes
Yes	No		Always	Most of the times	Most of the times	Sometimes	Never	Never	Never	Never	Never	Sometimes	Never
Yes	No		Always	Most of the times	Most of the times	Sometimes	Never	Never	Never	Never	Never	Always	Sometimes
No	No		Always	Always	Most of the times	Sometimes	Never	Never	Never	Never	Never	Most of the times	Sometimes
No	No		Always	Always	Sometimes	Sometimes	Never	Never	Never	Never	Never	Most of the times	Sometimes
No	No		Most of the times	Most of the times	Sometimes	Sometimes	Never	Never	Never	Never	Never	Most of the times	Sometimes
Yes	No		Most of the times	Most of the times	Sometimes	Sometimes	Never	Never	Never	Never	Never	Sometimes	Never
No	No		Always	Always	Most of the times	Most of the times	Never	Never	Never	Never	Never	Always	Never
No	No		Always	Most of the times	Most of the times	Most of the times	Never	Never	Never	Never	Never	Most of the times	Never
Yes	No		Always	Most of the times	Sometimes	Sometimes	Never	Never	Never	Never	Never	Most of the times	Sometimes
No	No		Most of the times	Most of the times	Most of the times	Most of the times	Never	Never	Never	Never	Never	Most of the times	Never
No	No		Most of the times	Most of the times	Most of the times	Most of the times	Sometimes	Never	Never	Never	Never	Most of the times	Never
No	No		Most of the times	Most of the times	Sometimes	Sometimes	Never	Never	Never	Never	Never	Sometimes	Never
No	No		Always	Most of the times	Sometimes	Sometimes	Never	Never	Never	Never	Never	Sometimes	Sometimes
Yes	No		Always	Always	Most of the times	Never	Never	Never	Never	Never	Never	Most of the times	Sometimes
Yes	No		Always	Always	Most of the times	Sometimes	Never	Never	Never	Never	Never	Sometimes	Sometimes
Yes	No		Always	Always	Always	Most of the times	Sometimes	Never	Never	Never	Never	Most of the times	Sometimes
Yes	No		Always	Always	Most of the times	Most of the times	Most of the times	Never	Never	Never	Never	Most of the times	Always
Yes	No		Most of the times	Most of the times	Most of the times	Always	Most of the times	Never	Never	Sometimes	Never	Most of the times	Sometimes
Yes	No		Most of the times	Always	Most of the times	Most of the times	Never	Never	Never	Never	Never	Most of the times	Most of the times
Yes	No		Most of the times	Always	Most of the times	Never	Sometimes	Never	Never	Sometimes	Never	Most of the times	Sometimes
Yes	No		Always	Always	Always	Sometimes	Sometimes	Never	Never	Sometimes	Never	Most of the times	Sometimes
Yes	No		Always	Always	Most of the times	Always	Most of the times	Never	Always	Always	Never	Always	Always
No	No		Always	Always	Always	Always	Most of the times	Most of the times	Always	Most of the times	Never	Most of the times	Always
No	No		Most of the times	Always	Always	Always	Most of the times	Most of the times	Always	Always	Sometimes	Always	Always
Yes	No		Most of the times	Most of the times	Most of the times	Most of the times	Never	Never	Never	Never	Never	Most of the times	Most of the times
Yes	No		Always	Most of the times	Most of the times	Most of the times	Sometimes	Never	Never	Never	Never	Always	Most of the times
Yes	No		Most of the times	Most of the times	Sometimes	Most of the times	Most of the times	Never	Never	Never	Never	Most of the times	Sometimes
Yes	No		Most of the times	Most of the times	Sometimes	Never	Sometimes	Never	Never	Sometimes	Never	Most of the times	Most of the times
Yes	No		Most of the times	Most of the times	Most of the times	Sometimes	Never	Never	Never	Never	Never	Most of the times	Most of the times

Have you ever used the same letters of recommendation for different people?	Have you ever copied a letter of recommendation?	Where did you copy the letter(s) of recommendation from?	Your academic background and years of experience (Do you include the following in your letters of recommendation?)	The nature of your relationship with the applicant (Do you include the following in your letters of recommendation?)	The duration of your relationship with the applicant (Do you include the following in your letters of recommendation?)	The applicant's qualifications/suitability for the position (Do you include the following in your letters of recommendation?)	The applicant's research involvement (Do you include the following in your letters of recommendation?)	The applicant's extracurricular activities (Do you include the following in your letters of recommendation?)	The applicant's involvement in education (Do you include the following in your letters of recommendation?)	Specific examples of the applicant's abilities and traits (Do you include the following in your letters of recommendation?)	Areas for improvement for the applicant (Do you include the following in your letters of recommendation?)	Global assessment of applicant (as compared to other applicants) (Do you include the following in your letters of recommendation?)	Work ethic (Do you comment on the following in letters of recommendations?)
No	No		Always	Always	Always	Always	Sometimes	Sometimes	Sometimes	Always	Sometimes	Always	Always
Yes	No		Sometimes	Sometimes	Sometimes	Sometimes	Sometimes	Never	Never	Never	Never	Never	Most of the times
Yes	No		Sometimes	Sometimes	Sometimes	Sometimes	Sometimes	Never	Never	Never	Never	Never	Sometimes
Yes	Yes	From an online resource	Sometimes	Sometimes	Never	Sometimes	Never	Never	Never	Never	Never	Most of the times	Most of the times
No	No		Sometimes	Sometimes	Never	Sometimes	Never	Never	Never	Never	Never	Most of the times	Most of the times
No	No		Sometimes	Sometimes	Sometimes	Always	Sometimes	Never	Never	Never	Never	Sometimes	Sometimes
No	No		Never	Always	Always	Sometimes	Sometimes	Sometimes	Sometimes	Most of the times	Never	Never	Always
No	No		Always	Always	Always	Always	Always	Always	Always	Always	Most of the times	Most of the times	Always
Yes	No		Sometimes	Most of the times	Sometimes	Sometimes	Never	Never	Never	Never	Never	Sometimes	Most of the times
No	No		Sometimes	Most of the times	Always	Always	Always	Always	Always	Most of the times	Sometimes	Sometimes	Always
Yes	No		Never	Always	Always	Most of the times	Sometimes	Never	Sometimes	Sometimes	Never	Most of the times	Most of the times
No	No		Most of the times	Most of the times	Most of the times	Most of the times	Most of the times	Most of the times	Most of the times	Most of the times	Most of the times	Most of the times	Most of the times
Yes	No		Never	Always	Most of the times	Always	Always	Always	Always	Most of the times	Sometimes	Most of the times	Always
Yes	No		Most of the times	Always	Most of the times	Always	Most of the times	Most of the times	Sometimes	Always	Never	Most of the times	Always
No	No		Never	Always	Always	Always	Sometimes	Never	Sometimes	Always	Sometimes	Always	Most of the times
No	No		Sometimes	Always	Always	Always	Always	Sometimes	Always	Most of the times	Sometimes	Always	Always
No	No		Never	Most of the times	Most of the times	Sometimes	Most of the times	Sometimes	Most of the times	Most of the times	Sometimes	Sometimes	Most of the times
No	No		Never	Always	Always	Always	Most of the times	Always	Always	Always	Sometimes	Always	Always
Yes	No		Never	Always	Most of the times	Always	Most of the times	Never	Most of the times	Most of the times	Most of the times	Always	Most of the times
No	No		Always	Always	Always	Always	Always	Always	Most of the times	Always	Never	Most of the times	Always
No	No		Always	Always	Always	Most of the times	Most of the times	Sometimes	Sometimes	Sometimes	Never	Most of the times	Always
No	No		Never	Never	Always	Always	Sometimes	Sometimes	Sometimes	Never	Never	Never	Most of the times
No	No		Always	Always	Always	Never	Always	Never	Always	Always	Never	Never	Always
No	No		Always	Most of the times	Always	Always	Always	Always	Always	Always	Always	Always	Always
Yes	No		Always	Always	Always	Always	Never	Never	Never	Always	Never	Always	Always
No	No		Never	Always	Always	Always	Sometimes	Never	Sometimes	Most of the times	Sometimes	Always	Most of the times

Have you ever used the same letters of recommendation for different people?	Have you ever copied a letter of recommendation?	Where did you copy the letter(s) of recommendation from?	Your academic background and years of experience (Do you include the following in your letters of recommendation?)	The nature of your relationship with the applicant (Do you include the following in your letters of recommendation?)	The duration of your relationship with the applicant (Do you include the following in your letters of recommendation?)	The applicant's qualifications/suitability for the position (Do you include the following in your letters of recommendation?)	The applicant's research involvement (Do you include the following in your letters of recommendation?)	The applicant's extracurricular activities (Do you include the following in your letters of recommendation?)	The applicant's involvement in education (Do you include the following in your letters of recommendation?)	Specific examples of the applicant's abilities and traits (Do you include the following in your letters of recommendation?)	Areas for improvement for the applicant (Do you include the following in your letters of recommendation?)	Global assessment of applicant (as compared to other applicants) (Do you include the following in your letters of recommendation?)	Work ethic (Do you comment on the following in letters of recommendations?)
No	No		Sometimes	Always	Always	Always	Most of the times	Most of the times	Always	Always	Sometimes	Sometimes	Always
No	No		Always	Always	Always	Always	Always	Always	Always	Always	Always	Always	Always
Yes	No		Sometimes	Sometimes	Never	Most of the times	Never	Never	Never	Sometimes	Never	Most of the times	Sometimes
No	No		Never	Never	Never	Never	Never	Never	Never	Never	Never	Never	Never
No	No		Never	Always	Always	Always	Always	Never	Most of the times	Always	Never	Sometimes	Sometimes
No	No		Sometimes	Sometimes	Never	Most of the times	Never	Never	Never	Sometimes	Never	Sometimes	Sometimes
No	No		Never	Most of the times	Most of the times	Sometimes	Most of the times	Most of the times	Most of the times	Most of the times	Never	Never	Sometimes
No	No		Never	Never	Never	Never	Never	Never	Never	Never	Never	Never	Never
No	No		Most of the times	Always	Always	Most of the times	Most of the times	Most of the times	Most of the times	Most of the times	Never	Sometimes	Most of the times
No	No		Always	Always	Always	Always	Sometimes	Sometimes	Most of the times	Always	Always	Always	Always
No	Yes	From an online resource	Never	Always	Always	Most of the times	Most of the times	Never	Never	Most of the times	Never	Never	Always
No	No		Sometimes	Always	Always	Always	Always	Most of the times	Always	Always	Sometimes	Sometimes	Always
No	No		Sometimes	Most of the times	Most of the times	Most of the times	Sometimes	Sometimes	Sometimes	Sometimes	Sometimes	Most of the times	Sometimes
No	No		Sometimes	Always	Always	Always	Most of the times	Always	Most of the times	Always	Never	Always	Most of the times
No	No		Sometimes	Always	Always	Always	Always	Always	Always	Always	Never	Always	Most of the times
Yes	No		Never	Always	Always	Most of the times	Never	Sometimes	Sometimes	Always	Sometimes	Always	Always
Yes	No		Sometimes	Always	Sometimes	Always	Sometimes	Sometimes	Never	Never	Never	Always	Always
No	No		Sometimes	Always	Always	Always	Sometimes	Sometimes	Sometimes	Sometimes	Never	Sometimes	Sometimes
No	No		Sometimes	Always	Always	Always	Sometimes	Sometimes	Sometimes	Most of the times	Sometimes	Sometimes	Most of the times
No	No		Never	Always	Always	Always	Always	Sometimes	Sometimes	Most of the times	Never	Most of the times	Always
Yes	No		Most of the times	Always	Always	Sometimes	Most of the times	Most of the times	Always	Always	Never	Never	Always
No	No		Never	Always	Always	Always	Most of the times	Most of the times	Most of the times	Most of the times	Sometimes	Sometimes	Most of the times
No	No		Sometimes	Always	Always	Always	Sometimes	Never	Always	Most of the times	Sometimes	Always	Always
No	No		Sometimes	Most of the times	Most of the times	Most of the times	Most of the times	Most of the times	Most of the times	Most of the times	Most of the times	Most of the times	Most of the times
No	No		Most of the times	Always	Always	Sometimes	Sometimes	Never	Most of the times	Sometimes	Sometimes	Most of the times	Sometimes
No	No		Never	Always	Always	Always	Always	Always	Always	Always	Most of the times	Always	Always

Have you ever used the same letters of recommendation for different people?	Have you ever copied a letter of recommendation?	Where did you copy the letter(s) of recommendation from?	Your academic background and years of experience (Do you include the following in your letters of recommendation?)	The nature of your relationship with the applicant (Do you include the following in your letters of recommendation?)	The duration of your relationship with the applicant (Do you include the following in your letters of recommendation?)	The applicant's qualifications/suitability for the position (Do you include the following in your letters of recommendation?)	The applicant's research involvement (Do you include the following in your letters of recommendation?)	The applicant's extracurricular activities (Do you include the following in your letters of recommendation?)	The applicant's involvement in education (Do you include the following in your letters of recommendation?)	Specific examples of the applicant's abilities and traits (Do you include the following in your letters of recommendation?)	Areas for improvement for the applicant (Do you include the following in your letters of recommendation?)	Global assessment of applicant (as compared to other applicants) (Do you include the following in your letters of recommendation?)	Work ethic (Do you comment on the following in letters of recommendations?)
No	No		Never	Always	Always	Always	Always	Always	Always	Always	Always	Always	Always
No	No		Never	Always	Always	Always	Always	Always	Always	Sometimes	Never	Never	Most of the times
No	No		Never	Always	Always	Most of the times	Most of the times	Most of the times	Most of the times	Most of the times	Most of the times	Most of the times	Most of the times
No	No		Never	Most of the times	Sometimes	Most of the times	Most of the times	Sometimes	Always	Most of the times	Sometimes	Most of the times	Most of the times
Yes	No		Never	Always	Always	Always	Most of the times	Most of the times	Most of the times	Most of the times	Sometimes	Sometimes	Sometimes
No	No		Always	Always	Always	Always	Always	Always	Always	Always	Most of the times	Always	Always
No	No		Always	Always	Most of the times	Always	Always	Always	Always	Always	Sometimes	Most of the times	Sometimes
No	No		Always	Always	Always	Always	Most of the times	Sometimes	Always	Most of the times	Most of the times	Most of the times	Always
Yes	No		Sometimes	Always	Sometimes	Most of the times	Never	Never	Never	Never	Never	Always	Sometimes
No	No		Most of the times	Most of the times	Most of the times	Always	Most of the times	Most of the times	Sometimes	Sometimes	Sometimes	Most of the times	Most of the times
Yes	No		Never	Always	Most of the times	Always	Sometimes	Never	Sometimes	Never	Sometimes	Most of the times	Always
No	No		Most of the times	Always	Always	Always	Sometimes	Sometimes	Most of the times	Always	Never	Sometimes	Always
No	No		Most of the times	Most of the times	Most of the times	Most of the times	Most of the times	Never	Sometimes	Most of the times	Never	Most of the times	Most of the times
No	No		Never	Always	Always	Most of the times	Sometimes	Sometimes	Sometimes	Always	Never	Never	Always
Yes	Yes	From a colleague	Sometimes	Most of the times	Most of the times	Never	Most of the times	Never	Most of the times	Most of the times	Always	Never	Most of the times

Intellectual curiosity (Do you comment on the following in letters of recommendations?)	Medical knowledge (Do you comment on the following in letters of recommendations?)	Procedural skills (Do you comment on the following in letters of recommendations?)	Problem solving and patient management (Do you comment on the following in letters of recommendations?)	Behavioral and attitudinal Skills (Do you comment on the following in letters of recommendations?)	Communication skills (Do you comment on the following in letters of recommendations?)	Ability to work in team (Do you comment on the following in letters of recommendations?)	Leadership (Do you comment on the following in letters of recommendations?)	Motivation (Do you comment on the following in letters of recommendations?)	Sense of responsibility (Do you comment on the following in letters of recommendations?)	Patient advocacy (Do you comment on the following in letters of recommendations?)	Are you aware of standardized letters of recommendation (standardized letters of evaluation [SLOE] or structured evaluative letter [SEL])?
Most of the times	Most of the times	Most of the times	Most of the times	Always	Always	Always	Most of the times	Most of the times	Most of the times	Most of the times	No
Never	Always	Always	Always	Always	Always	Sometimes	Sometimes	Never	Never	Never	No
Never	Always	Never	Always	Sometimes	Most of the times	Sometimes	Sometimes	Never	Never	Never	
Never	Always	Never	Always	Always	Always	Sometimes	Sometimes	Never	Never	Never	No
Never	Always	Never	Always	Always	Always	Never	Sometimes	Never	Never	Never	No
Never	Always	Always	Always	Sometimes	Sometimes	Sometimes	Sometimes	Never	Never	Never	No
Never	Always	Always	Always	Always	Always	Sometimes	Sometimes	Never	Never	Never	Yes
Never	Always	Always	Always	Always	Always	Never	Sometimes	Never	Never	Never	Yes
Never	Always	Always	Always	Always	Always	Never	Sometimes	Never	Never	Never	Yes
Never	Always	Always	Always	Always	Always	Sometimes	Sometimes	Never	Never	Never	No
Never	Always	Always	Always	Always	Always	Never	Sometimes	Never	Never	Never	No
Never	Always	Always	Always	Always	Always	Sometimes	Sometimes	Never	Never	Never	No
Never	Always	Always	Always	Always	Always	Never	Sometimes	Never	Never	Never	Yes
Never	Always	Always	Always	Always	Always	Never	Sometimes	Never	Never	Never	No
Sometimes	Always	Always	Always	Always	Always	Sometimes	Sometimes	Never	Never	Never	No
Never	Always	Always	Always	Always	Always	Most of the times	Most of the times	Never	Never	Never	No
Never	Always	Always	Always	Always	Always	Sometimes	Sometimes	Never	Never	Never	No
Never	Always	Always	Always	Always	Always	Never	Never	Never	Never	Never	No
Never	Always	Always	Always	Always	Always	Sometimes	Sometimes	Never	Never	Never	No
Never	Always	Always	Always	Always	Always	Never	Sometimes	Never	Never	Never	No
Never	Always	Always	Always	Always	Always	Never	Sometimes	Never	Never	Never	No
Never	Always	Always	Always	Always	Always	Never	Sometimes	Never	Never	Never	No
Never	Always	Always	Always	Always	Always	Never	Most of the times	Never	Never	Never	No
Sometimes	Always	Always	Always	Always	Always	Sometimes	Sometimes	Never	Never	Never	No
Sometimes	Always	Always	Always	Always	Always	Sometimes	Sometimes	Never	Never	Never	No
Sometimes	Always	Always	Always	Always	Always	Sometimes	Sometimes	Never	Never	Never	No
Sometimes	Always	Always	Always	Always	Always	Sometimes	Sometimes	Never	Never	Never	No
Always	Always	Sometimes	Always	Always	Always	Always	Sometimes	Always	Always	Always	No
Always	Always	Always	Sometimes	Sometimes	Most of the times	Most of the times	Most of the times	Most of the times	Most of the times	Most of the times	No
Always	Always	Most of the times	Always	Always	Always	Always	Most of the times	Always	Most of the times	Most of the times	No
Never	Always	Always	Always	Always	Always	Sometimes	Sometimes	Never	Never	Never	No
Sometimes	Always	Always	Always	Always	Always	Sometimes	Sometimes	Never	Never	Never	No
Never	Always	Always	Always	Always	Always	Never	Never	Never	Never	Never	No
Sometimes	Always	Always	Always	Always	Always	Never	Sometimes	Never	Never	Never	No
Never	Always	Always	Always	Always	Always	Sometimes	Sometimes	Never	Never	Never	No

Intellectual curiosity (Do you comment on the following in letters of recommendations?)	Medical knowledge (Do you comment on the following in letters of recommendations?)	Procedural skills (Do you comment on the following in letters of recommendations?)	Problem solving and patient management (Do you comment on the following in letters of recommendations?)	Behavioral and attitudinal Skills (Do you comment on the following in letters of recommendations?)	Communication skills (Do you comment on the following in letters of recommendations?)	Ability to work in team (Do you comment on the following in letters of recommendations?)	Leadership (Do you comment on the following in letters of recommendations?)	Motivation (Do you comment on the following in letters of recommendations?)	Sense of responsibility (Do you comment on the following in letters of recommendations?)	Patient advocacy (Do you comment on the following in letters of recommendations?)	Are you aware of standardized letters of recommendation (standardized letters of evaluation [SLOE] or structured evaluative letter [SEL])?
Always	Always	Always	Always	Always	Always	Always	Sometimes	Sometimes	Sometimes	Always	No
Sometimes	Always	Always	Always	Always	Always	Sometimes	Sometimes	Never	Never	Never	
Sometimes	Always	Always	Always	Always	Always	Sometimes	Sometimes	Never	Never	Never	No
Sometimes	Always	Always	Always	Always	Most of the times	Never	Never	Never	Never	Never	No
Never	Always	Always	Always	Always	Always	Sometimes	Sometimes	Never	Never	Never	No
Never	Always	Most of the times	Always	Most of the times	Most of the times	Sometimes	Never	Never	Never	Never	No
Sometimes	Most of the times	Sometimes	Sometimes	Sometimes	Most of the times	Sometimes	Never	Always	Always	Never	No
Always	Always	Always	Always	Always	Always	Always	Always	Always	Always	Always	Yes
Never	Always	Always	Always	Always	Always	Sometimes	Sometimes	Never	Sometimes	Never	No
Sometimes	Most of the times	Most of the times	Sometimes	Most of the times	Most of the times	Most of the times	Sometimes	Most of the times	Sometimes	Never	No
Always	Sometimes	Sometimes	Sometimes	Always	Always	Always	Sometimes	Most of the times	Most of the times	Sometimes	No
Most of the times	Most of the times	Most of the times	Most of the times	Most of the times	Most of the times	Most of the times	Most of the times	Most of the times	Most of the times	Most of the times	No
Always	Always	Always	Always	Always	Always	Always	Always	Always	Always	Always	No
Always	Always	Sometimes	Most of the times	Always	Always	Always	Sometimes	Most of the times	Always	Sometimes	No
Most of the times	Always	Never	Most of the times	Always	Always	Always	Sometimes	Always	Always	Always	No
Always	Always	Sometimes	Most of the times	Always	Most of the times	Most of the times	Most of the times	Always	Always	Sometimes	No
Most of the times	Most of the times	Most of the times	Most of the times	Never	Most of the times	Most of the times	Most of the times	Most of the times	Most of the times	Most of the times	No
Most of the times	Always	Always	Most of the times	Always	Most of the times	Always	Always	Most of the times	Always	Sometimes	Yes
Most of the times	Always	Always	Most of the times	Always	Always	Always	Most of the times	Always	Always	Sometimes	No
Always	Always	Sometimes	Always	Always	Most of the times	Most of the times	Most of the times	Always	Always	Always	Yes
Always	Always	Sometimes	Most of the times	Always	Always	Always	Sometimes	Always	Always	Sometimes	No
Most of the times	Always	Sometimes	Most of the times	Most of the times	Most of the times	Most of the times	Sometimes	Most of the times	Most of the times	Most of the times	No
Always	Most of the times	Never	Most of the times	Always	Sometimes	Always	Sometimes	Always	Always	Sometimes	No
Always	Always	Most of the times	Always	Most of the times	Always	Always	Most of the times	Always	Always	Most of the times	No
Always	Always	Always	Always	Always	Always	Always	Most of the times	Always	Always	Most of the times	Yes
Most of the times	Most of the times	Most of the times	Most of the times	Most of the times	Most of the times	Most of the times	Sometimes	Most of the times	Most of the times	Most of the times	No

Intellectual curiosity (Do you comment on the following in letters of recommendations?)	Medical knowledge (Do you comment on the following in letters of recommendations?)	Procedural skills (Do you comment on the following in letters of recommendations?)	Problem solving and patient management (Do you comment on the following in letters of recommendations?)	Behavioral and attitudinal Skills (Do you comment on the following in letters of recommendations?)	Communication skills (Do you comment on the following in letters of recommendations?)	Ability to work in team (Do you comment on the following in letters of recommendations?)	Leadership (Do you comment on the following in letters of recommendations?)	Motivation (Do you comment on the following in letters of recommendations?)	Sense of responsibility (Do you comment on the following in letters of recommendations?)	Patient advocacy (Do you comment on the following in letters of recommendations?)	Are you aware of standardized letters of recommendation (standardized letters of evaluation [SLOE] or structured evaluative letter [SEL])?
Always	Always	Sometimes	Always	Always	Always	Always	Always	Always	Always	Sometimes	No
Always	Always	Always	Always	Always	Always	Always	Always	Always	Always	Always	Yes
Never	Always	Always	Always	Most of the times	Most of the times	Sometimes	Never	Never	Never	Never	No
Never	Never	Never	Never	Never	Never	Never	Never	Never	Never	Never	No
Sometimes	Always	Always	Sometimes	Sometimes	Sometimes	Sometimes	Sometimes	Sometimes	Sometimes	Sometimes	No
Never	Always	Always	Always	Always	Always	Never	Never	Never	Never	Never	No
Most of the times	Sometimes	Sometimes	Sometimes	Sometimes	Most of the times	Most of the times	Most of the times	Most of the times	Most of the times	Most of the times	No
Never	Never	Never	Never	Never	Never	Never	Never	Never	Never	Never	No
Most of the times	Most of the times	Sometimes	Most of the times	Most of the times	Always	Always	Most of the times	Always	Most of the times	Sometimes	Yes
Sometimes	Always	Always	Always	Most of the times	Always	Always	Always	Sometimes	Most of the times	Always	No
Never	Always	Always	Most of the times	Always	Always	Always	Sometimes	Most of the times	Most of the times	Most of the times	No
Most of the times	Always	Always	Most of the times	Always	Always	Always	Most of the times	Sometimes	Most of the times	Most of the times	Yes
Sometimes	Most of the times	Most of the times	Most of the times	Sometimes	Most of the times	Most of the times	Sometimes	Most of the times	Sometimes	Sometimes	No
Sometimes	Always	Sometimes	Most of the times	Always	Always	Always	Most of the times	Most of the times	Most of the times	Sometimes	Yes
Most of the times	Most of the times	Sometimes	Sometimes	Most of the times	Most of the times	Most of the times	Most of the times	Most of the times	Most of the times	Most of the times	
Never	Always	Most of the times	Always	Always	Always	Always	Never	Most of the times	Always	Never	No
Never	Always	Always	Always	Always	Always	Never	Never	Never	Never	Never	No
Sometimes	Sometimes	Sometimes	Sometimes	Sometimes	Sometimes	Sometimes	Sometimes	Sometimes	Sometimes	Sometimes	Yes
Most of the times	Always	Sometimes	Sometimes	Always	Most of the times	Most of the times	Sometimes	Most of the times	Most of the times	Sometimes	No
Always	Always	Most of the times	Most of the times	Always	Sometimes	Most of the times	Sometimes	Most of the times	Most of the times	Sometimes	No
Most of the times	Most of the times	Most of the times	Never	Always	Sometimes	Most of the times	Most of the times	Always	Always	Most of the times	No
Most of the times	Most of the times	Most of the times	Most of the times	Most of the times	Most of the times	Most of the times	Sometimes	Most of the times	Most of the times	Sometimes	No
Most of the times	Always	Sometimes	Most of the times	Always	Always	Always	Most of the times	Most of the times	Most of the times	Sometimes	No
Most of the times	Most of the times	Most of the times	Most of the times	Most of the times	Most of the times	Most of the times	Most of the times	Most of the times	Most of the times	Most of the times	No
Most of the times	Most of the times	Most of the times	Most of the times	Sometimes	Most of the times	Most of the times	Most of the times	Most of the times	Most of the times	Sometimes	No
Always	Always	Always	Always	Always	Always	Always	Always	Always	Always	Sometimes	No

Intellectual curiosity (Do you comment on the following in letters of recommendations?)	Medical knowledge (Do you comment on the following in letters of recommendations?)	Procedural skills (Do you comment on the following in letters of recommendations?)	Problem solving and patient management (Do you comment on the following in letters of recommendations?)	Behavioral and attitudinal Skills (Do you comment on the following in letters of recommendations?)	Communication skills (Do you comment on the following in letters of recommendations?)	Ability to work in team (Do you comment on the following in letters of recommendations?)	Leadership (Do you comment on the following in letters of recommendations?)	Motivation (Do you comment on the following in letters of recommendations?)	Sense of responsibility (Do you comment on the following in letters of recommendations?)	Patient advocacy (Do you comment on the following in letters of recommendations?)	Are you aware of standardized letters of recommendation (standardized letters of evaluation [SLOE] or structured evaluative letter [SEL])?
Always	Always	Always	Always	Always	Always	Always	Always	Always	Always	Always	No
Most of the times	Always	Most of the times	Most of the times	Always	Always	Always	Most of the times	Always	Most of the times	Never	No
Most of the times	Always	Always	Always	Always	Always	Always	Most of the times	Sometimes	Most of the times	Most of the times	Yes
Most of the times	Always	Most of the times	Most of the times	Most of the times	Always	Always	Most of the times	Sometimes	Always	Always	No
Sometimes	Always	Sometimes	Sometimes	Most of the times	Most of the times	Most of the times	Most of the times	Most of the times	Most of the times	Sometimes	No
Sometimes	Most of the times	Sometimes	Always	Always	Always	Always	Always	Always	Always	Always	No
Most of the times	Most of the times	Sometimes	Most of the times	Most of the times	Most of the times	Most of the times	Most of the times	Most of the times	Most of the times	Most of the times	Yes
Always	Always	Always	Always	Always	Always	Always	Most of the times	Most of the times	Always	Always	Yes
Never	Always	Sometimes	Most of the times	Most of the times	Most of the times	Never	Never	Never	Never	Never	No
Most of the times	Always	Always	Always	Always	Always	Always	Sometimes	Sometimes	Sometimes	Most of the times	No
Sometimes	Most of the times	Sometimes	Most of the times	Always	Always	Always	Most of the times	Always	Always	Sometimes	Yes
Most of the times	Always	Always	Always	Always	Always	Always	Always	Always	Most of the times	Always	No
Most of the times	Most of the times	Sometimes	Sometimes	Most of the times	Most of the times	Most of the times	Sometimes	Most of the times	Most of the times	Sometimes	No
Never	Always	Most of the times	Most of the times	Sometimes	Always	Always	Sometimes	Sometimes	Sometimes	Sometimes	No
Sometimes	Most of the times	Sometimes	Always	Most of the times	Always	Most of the times	Always	Most of the times	Most of the times	Most of the times	

Are you aware of the residency programs that require standardized letters of recommendation?	Have you ever written a standardized letter of recommendation?	Have you ever received guidance/training in writing letters of recommendations?	Where did you receive the guidance/training?	Are you interested in receiving training on writing letters of recommendations?	Which of the following would be your preferred method?	Please feel free to leave us any additional comments
No	No	No		Yes	Enduring materials (eg. journal articles, text books)	
No	No	No		Yes	Webinar/Online course	
No	No	No		Yes	Webinar/Online course	
No	No	No		Yes	Webinar/Online course	
No	No	No		Yes	Webinar/Online course	
No	No	No		Yes	Webinar/Online course	
No	No	No		Yes	Webinar/Online course	
Yes	No	No		Yes	Webinar/Online course	
No	No	No		Yes	Webinar/Online course	
No	No	No		Yes	Webinar/Online course	
No	No	No		Yes	Webinar/Online course	
No	No	No		Yes	Webinar/Online course	
No	No	No		Yes	Webinar/Online course	
No	No	No		Yes	Webinar/Online course	
No	No	No		Yes	Webinar/Online course	
No	No	No		Yes	Webinar/Online course	
No	No	No		Yes	Webinar/Online course	
No	No	No		Yes	Webinar/Online course	
No	No	No		Yes	Webinar/Online course	
No	No	No		Yes	Webinar/Online course	
No	No	No		Yes	Webinar/Online course	
No	No	No		Yes	Webinar/Online course	
No	No	No		Yes	Webinar/Online course	
No	No	No		Yes	Webinar/Online course	
No	No	No		Yes	Webinar/Online course	
No	No	No		Yes	Webinar/Online course	
No	No	Yes	Faculty development/formal training at your institution	Yes	Webinar/Online course	
No	No	No		Yes	In-person lecture/course	
Yes	No	Yes	Discussions with program directors	Yes	Webinar/Online course	
No	No	No		Yes	Webinar/Online course	
No	No	No		Yes	Webinar/Online course	
No	No	No		Yes	Webinar/Online course	
No	No	No		Yes	Webinar/Online course	
No	No	No		Yes	Webinar/Online course	

Are you aware of the residency programs that require standardized letters of recommendation?	Have you ever written a standardized letter of recommendation?	Have you ever received guidance/training in writing letters of recommendations?	Where did you receive the guidance/training?	Are you interested in receiving training on writing letters of recommendations?	Which of the following would be your preferred method?	Please feel free to leave us any additional comments
Yes	Yes	No		Yes	In-person lecture/course	I often get asked to write letters by trainees to whom I have had very little exposure. I will frequently decline these unless they have proven to be exceptional as I have no firm basis to write these on
No	No	No		Yes	Webinar/Online course	
No	No	No		Yes	Webinar/Online course	
No	No	No		Yes	Webinar/Online course	
No	No	No		Yes	Webinar/Online course	
No	No	No		Yes	Webinar/Online course	
Yes	Yes	No		Yes	Webinar/Online course	
No	No	No		Yes	Webinar/Online course	
No	No	No		Yes	Webinar/Online course	
No	No	No		Yes	Webinar/Online course	
No	No	No		Yes	Webinar/Online course	
No	No	No		Yes	In-person lecture/course	
No	Yes	No		Yes	Webinar/Online course	
No	No	No		Yes	Webinar/Online course	
No	No	No		No		
No	No	No		Yes	Webinar/Online course	
Yes	Yes	Yes	Discussions with program directors	Yes	In-person lecture/course	
No	No	No		Yes	In-person lecture/course	
No	No	No		Yes	In-person lecture/course	
No	No	No		Yes	In-person lecture/course	
No	No	No		Yes	Webinar/Online course	
No	No	No		Yes	Webinar/Online course	
No	No	No		Yes	Webinar/Online course	
Yes	Yes	No		No		
No	No	No		No		

Are you aware of the residency programs that require standardized letters of recommendation?	Have you ever written a standardized letter of recommendation?	Have you ever received guidance/training in writing letters of recommendations?	Where did you receive the guidance/training?	Are you interested in receiving training on writing letters of recommendations?	Which of the following would be your preferred method?	Please feel free to leave us any additional comments
No	No	No		Yes	Webinar/Online course	
Yes	Yes	No		Yes	In-person lecture/course	
No	No	No		Yes	Webinar/Online course	
No	No	No		Yes	Webinar/Online course	
No	No	No		Yes	In-person lecture/course	
No	No	No		Yes	Webinar/Online course	
No	No	No		Yes	In-person lecture/course	
No	No	No		Yes	Webinar/Online course	
No	No	Yes	Conferences	Yes	Webinar/Online course	
No	No	No		Yes	Webinar/Online course	
No	No	No		Yes	Webinar/Online course	
No	No	No		No		
No	No	No		Yes	In-person lecture/course	
No	Yes	No		Yes	Webinar/Online course	
No	No	No		Yes	Webinar/Online course	
No	No	No		Yes	Webinar/Online course	
Yes	Yes	Yes	Other: please describe Discussion with mentors, colleagues, program directors	Yes	Webinar/Online course	The survey answers reflect my experience over my career.
No	No	No		Yes	In-person lecture/course	
No	No	No		Yes	Webinar/Online course	
No	No	No		No		
No	No	No		No		
No	No	No		Yes	Webinar/Online course	
No	No	No		Yes	In-person lecture/course	
No	No	Yes	Discussions with colleagues	Yes	Enduring materials (eg. journal articles, text books)	
No	No	No		Yes	Webinar/Online course	

Are you aware of the residency programs that require standardized letters of recommendation?	Have you ever written a standardized letter of recommendation?	Have you ever received guidance/training in writing letters of recommendations?	Where did you receive the guidance/training?	Are you interested in receiving training on writing letters of recommendations?	Which of the following would be your preferred method?	Please feel free to leave us any additional comments
No	No	No		Yes	Enduring materials (eg. journal articles, text books)	
No	No	No		Yes	In-person lecture/course	
Yes	Yes	Yes	Faculty development/formal training at your institution	No		
No	No	No		Yes	In-person lecture/course	
No	No	No		Yes	In-person lecture/course	
No	No	No		No		
No	No	No		Yes	Other, please describe Online course which is recorded with supplementary materials	
No	No	No		Yes	In-person lecture/course	
No	No	No		Yes	Webinar/Online course	
No	Yes	No		No		
Yes	Yes	No		No		
No	No	No		Yes	In-person lecture/course	
No	No	No		No		
No	No	No		No		
