# Supplementary material for: Components of effective letters of recommendation: A cross-sectional survey of academic faculty
Source: PLoS One. 2024 Jan 23;19(1):e0296637. doi: 10.1371/journal.pone.0296637 (PMC10805295; doi:10.1371/journal.pone.0296637)
Supplement: Attachment — Submitted filename: Response to reviewers.docx [file pone.0296637.s003.docx]

December 7th 2023

Dr. Amitav Banerjee, Academic Editor

PLOS ONE

Dear Dr. Amitav Banerjee,

Thank you for consideration of our manuscript. We appreciate the academic editor and reviewer’s efforts. Please see our point-by-point responses to comments below.

**Editor’s comment 1**. When submitting your revision, we need you to address these additional requirements.

Please ensure that your manuscript meets PLOS ONE's style requirements, including those for file naming. The PLOS ONE style templates can be found at https://journals.plos.org/plosone/s/file?id=wjVg/PLOSOne_formatting_sample_main_body.pdf and https://journals.plos.org/plosone/s/file?id=ba62/PLOSOne_formatting_sample_title_authors_affiliations.pdf

**Response:** The revised manuscript meets PLOS ONE style.

**Editor’s comment 2.** Did you know that depositing data in a repository is associated with up to a 25% citation advantage (https://doi.org/10.1371/journal.pone.0230416)? If you’ve not already done so, consider depositing your raw data in a repository to ensure your work is read, appreciated and cited by the largest possible audience. You’ll also earn an Accessible Data icon on your published paper if you deposit your data in any participating repository (<https://plos.org/open-science/open-data/#accessible-data>).

**Response:** Noted

**Editor’s comment 3**. We note that you have indicated that data from this study are available upon request. PLOS only allows data to be available upon request if there are legal or ethical restrictions on sharing data publicly. For more information on unacceptable data access restrictions, please see http://journals.plos.org/plosone/s/data-availability#loc-unacceptable-data-access-restrictions.

**Response:** The data set is now included with the manuscript as supplemental material.

In your revised cover letter, please address the following prompts:

a) If there are ethical or legal restrictions on sharing a de-identified dataset, please explain them in detail (e.g., data contain potentially sensitive information, data are owned by a third-party organization, etc.) and who has imposed them (e.g., an ethics committee). Please also provide contact information for a data access committee, ethics committee, or other institutional body to which data requests may be sent.

b) If there are no restrictions, please upload the minimal anonymized data set necessary to replicate your study findings as either Supporting Information files or to a stable, public repository and provide us with the relevant URLs, DOIs, or accession numbers. For a list of acceptable repositories, please see <http://journals.plos.org/plosone/s/data-availability#loc-recommended-repositories>.

**Response:** There are no legal or ethical restrictions. The datasets are included as a supplement 2 Appendix . The cover letter now highlights this point.

We will update your Data Availability statement on your behalf to reflect the information you provide.

**Editors comment 4**. We note that you have referenced Maruca-Sullivan PE, Lane CE, Moore EZ, Ross DA which has currently not yet been accepted for publication. Please remove this from your References and amend this to state in the body of your manuscript: Maruca-Sullivan PE, Lane CE, Moore EZ, Ross DA [Submitted]”) as detailed online in our guide for authors
http://journals.plos.org/plosone/s/submission-guidelines#loc-reference-style

**Response:** Please note that this reference has been published in Medical Education Journal in 2018. The reference is correct as written.

**Editors comment 5**. Please review your reference list to ensure that it is complete and correct. If you have cited papers that have been retracted, please include the rationale for doing so in the manuscript text, or remove these references and replace them with relevant current references. Any changes to the reference list should be mentioned in the rebuttal letter that accompanies your revised manuscript. If you need to cite a retracted article, indicate the article’s retracted status in the References list and also include a citation and full reference for the retraction notice.

**Response:** The reference list has been revised and I confirm that it is complete and correct in the revised manuscript version.
